# Supplementary figures and images for: Proteomic and functional comparison between human induced and embryonic stem cells
Source: eLife. 2024 Nov 14;13:RP92025. doi: 10.7554/eLife.92025 (PMC11563575; doi:10.7554/eLife.92025)

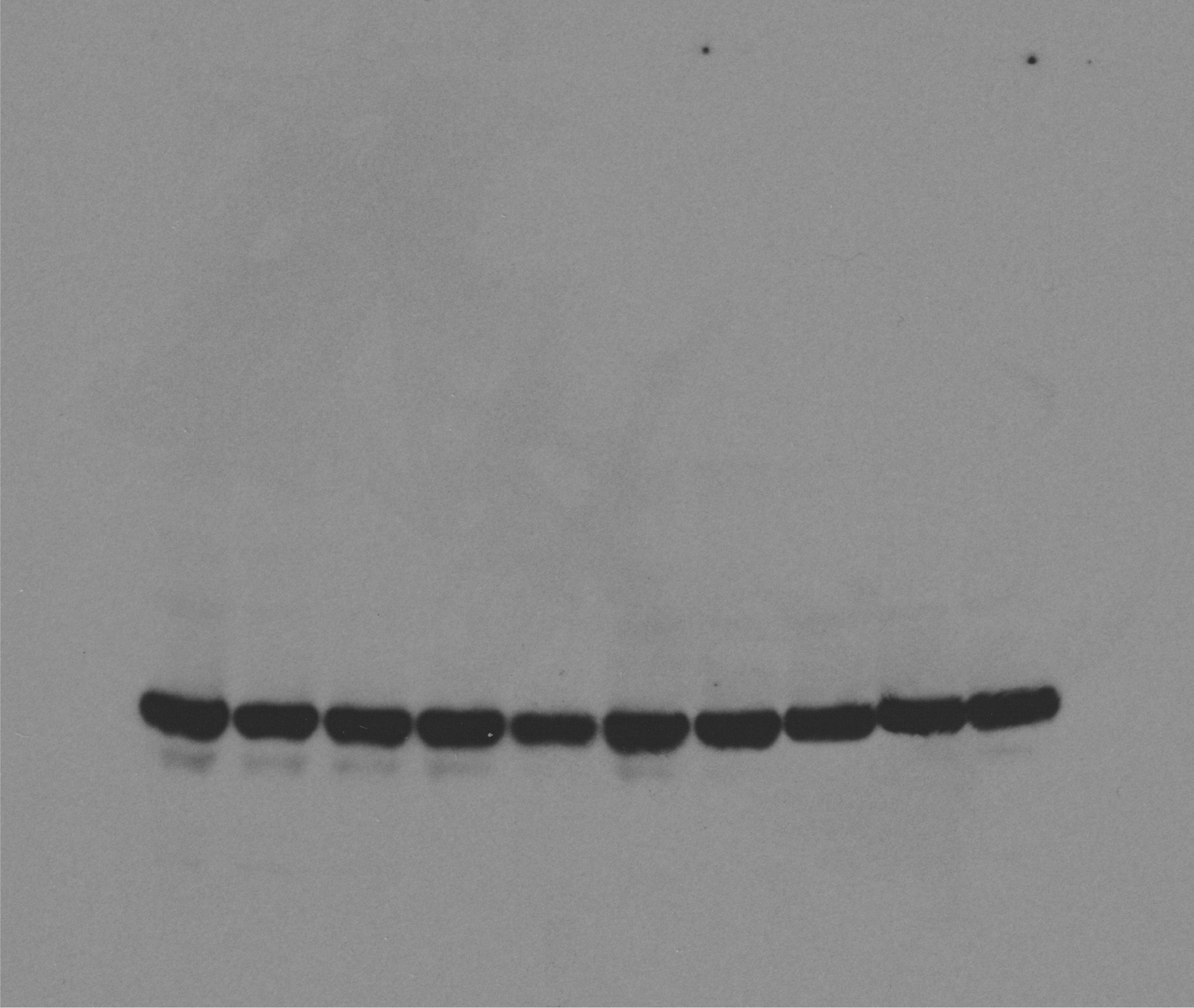

Supplement: Figure 1—source data 1. [file elife-92025-fig1-data1.zip › Figure 1-source data 1/NANOG WB.tif]

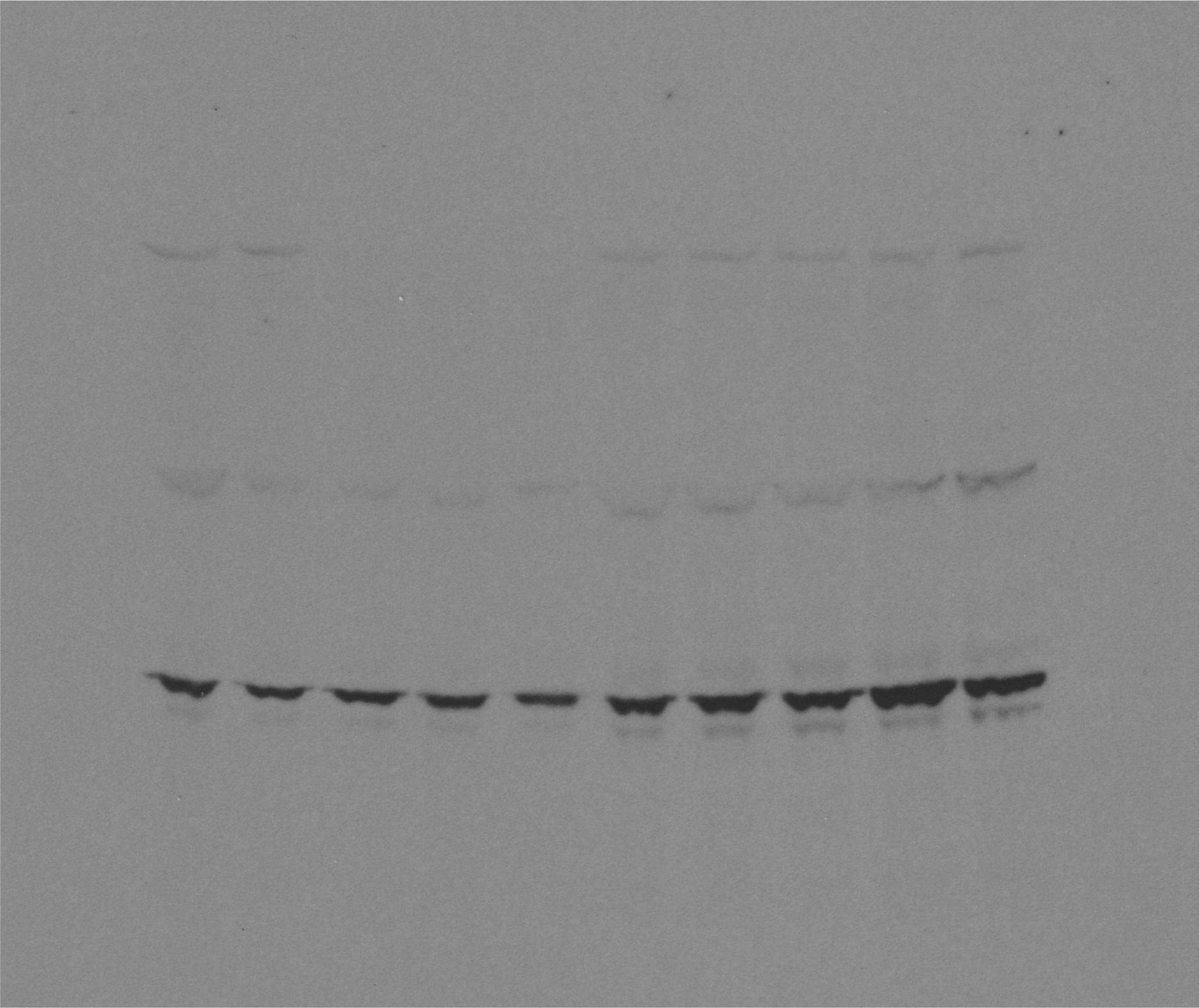

Supplement: Figure 1—source data 1. [file elife-92025-fig1-data1.zip › Figure 1-source data 1/GAPDH WB.tif]

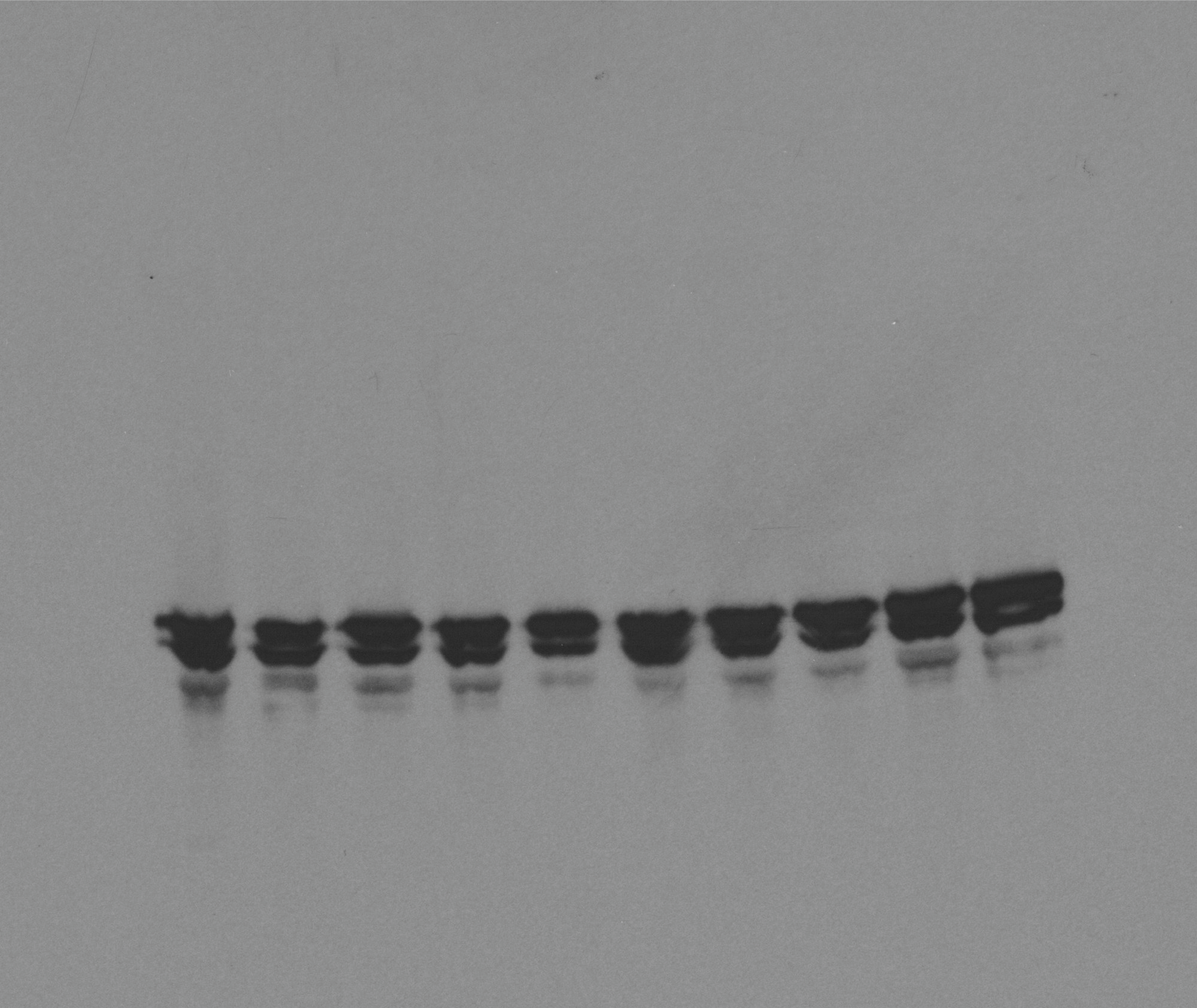

Supplement: Figure 1—source data 1. [file elife-92025-fig1-data1.zip › Figure 1-source data 1/OCT4 WB.tif]

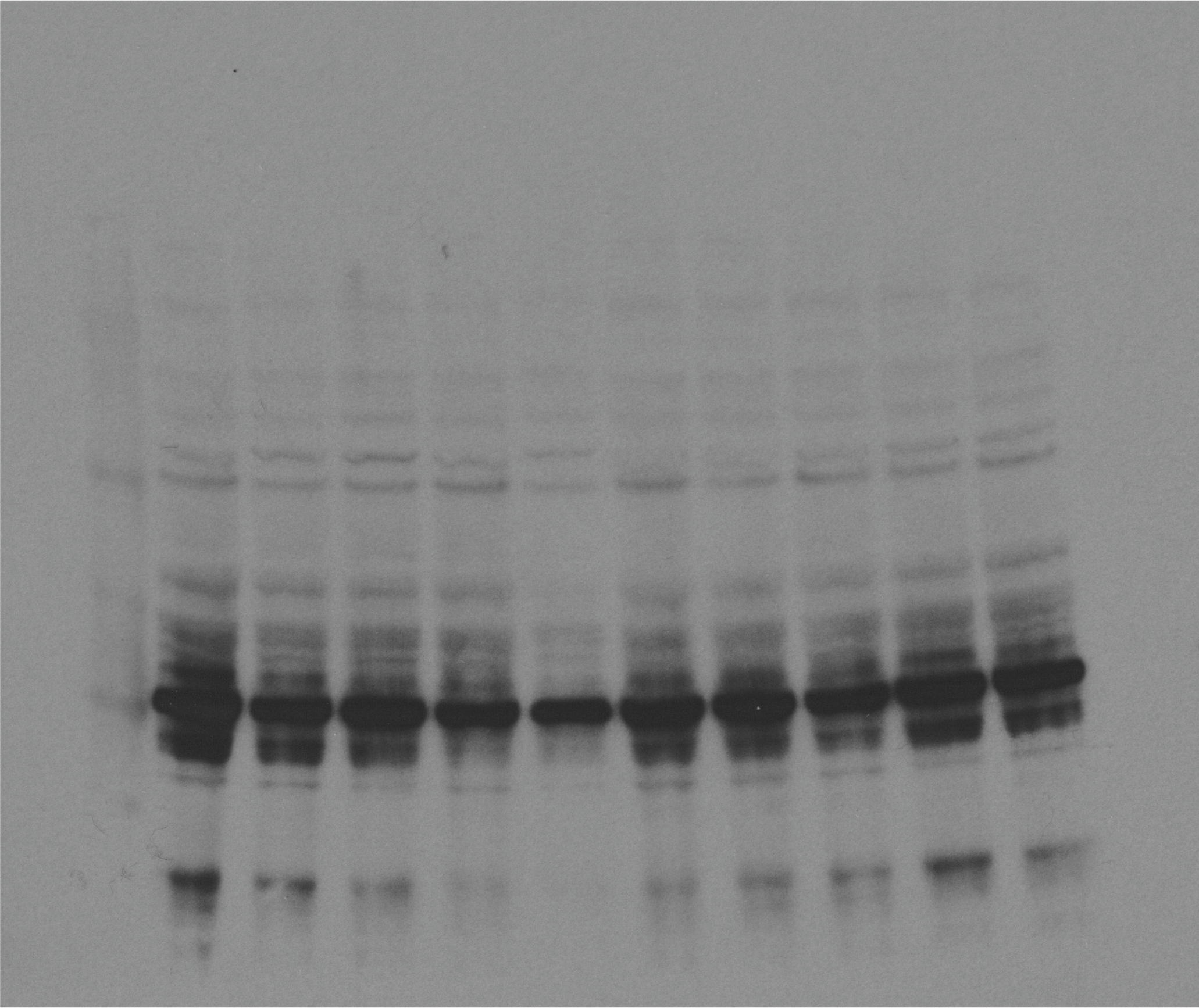

Supplement: Figure 1—source data 1. [file elife-92025-fig1-data1.zip › Figure 1-source data 1/SOX2 WB.tif]

**Figure 6i**

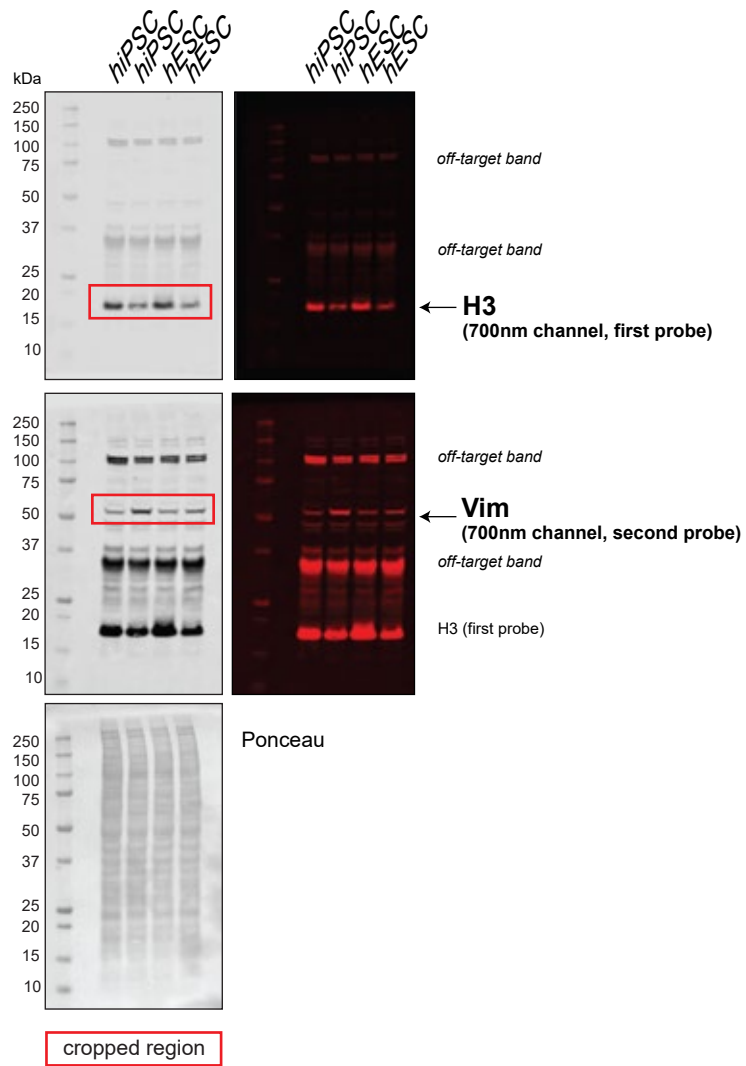

**Figure 6j**

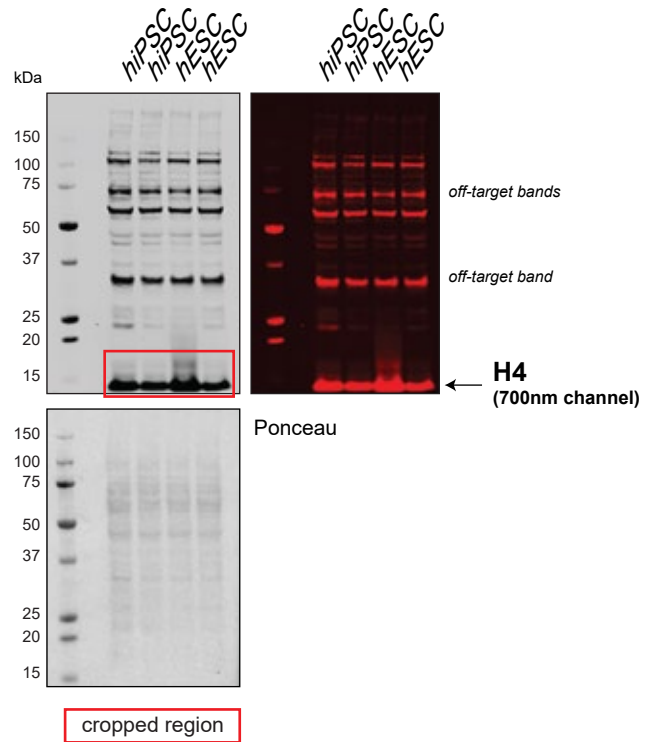

Supplement: Figure 6—source data 2. [file elife-92025-fig6-data2.zip › Figure 6i 6j-Source data 2 PDF file containing labelled uncropped western blots.pdf]
