# Supplementary material for: Proteomic and functional comparison between human induced and embryonic stem cells
Source: eLife. 2024 Nov 14;13:RP92025. doi: 10.7554/eLife.92025 (PMC11563575; doi:10.7554/eLife.92025)
Supplement: Figure 1—source data 2. [file elife-92025-fig1-data2.zip › Figure 1- source data 2.pdf]

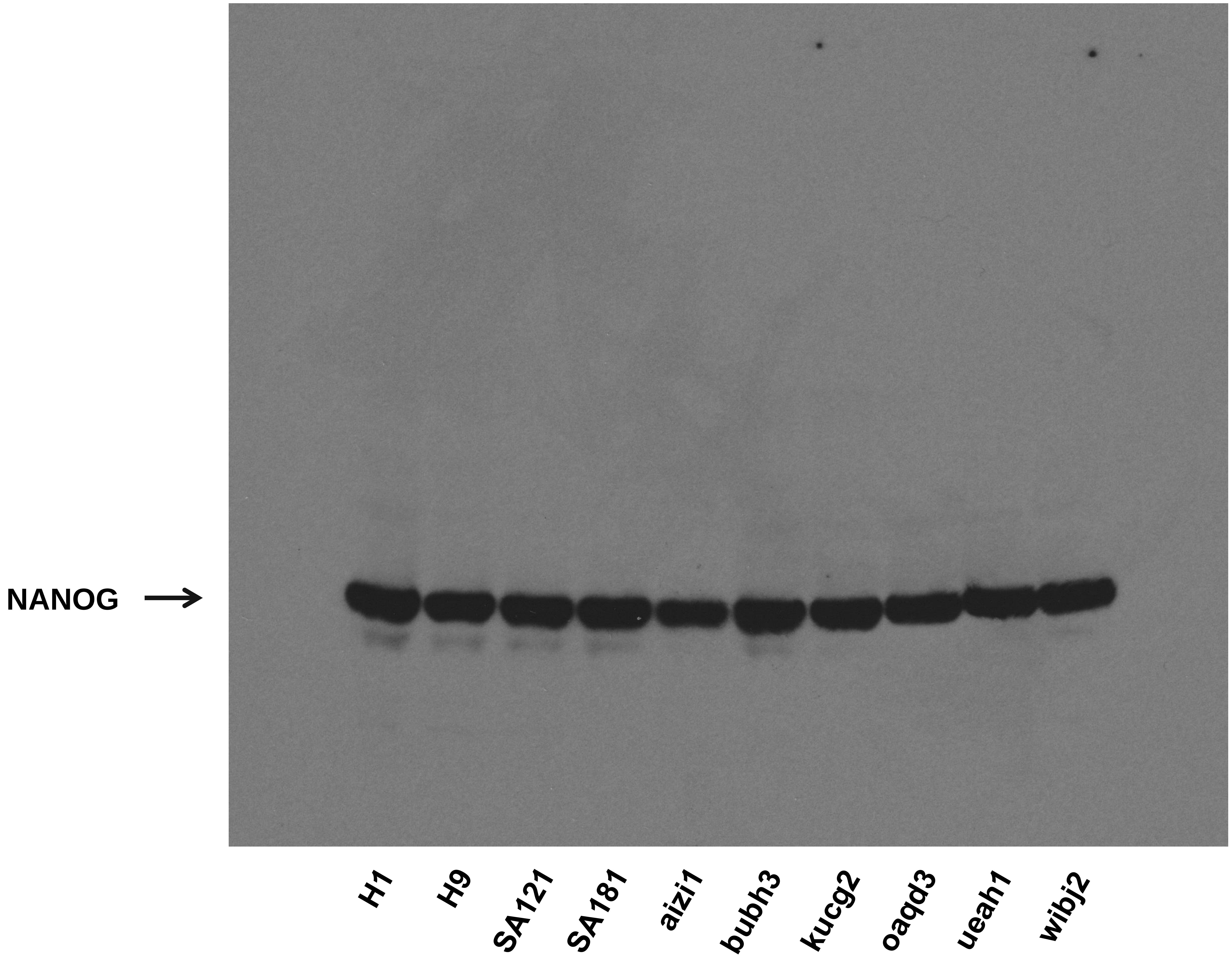
NANOG
H1
H9
SA121
SA181
aizi1
bubh3
kucg2
oaqd3
ueah1
wibj2

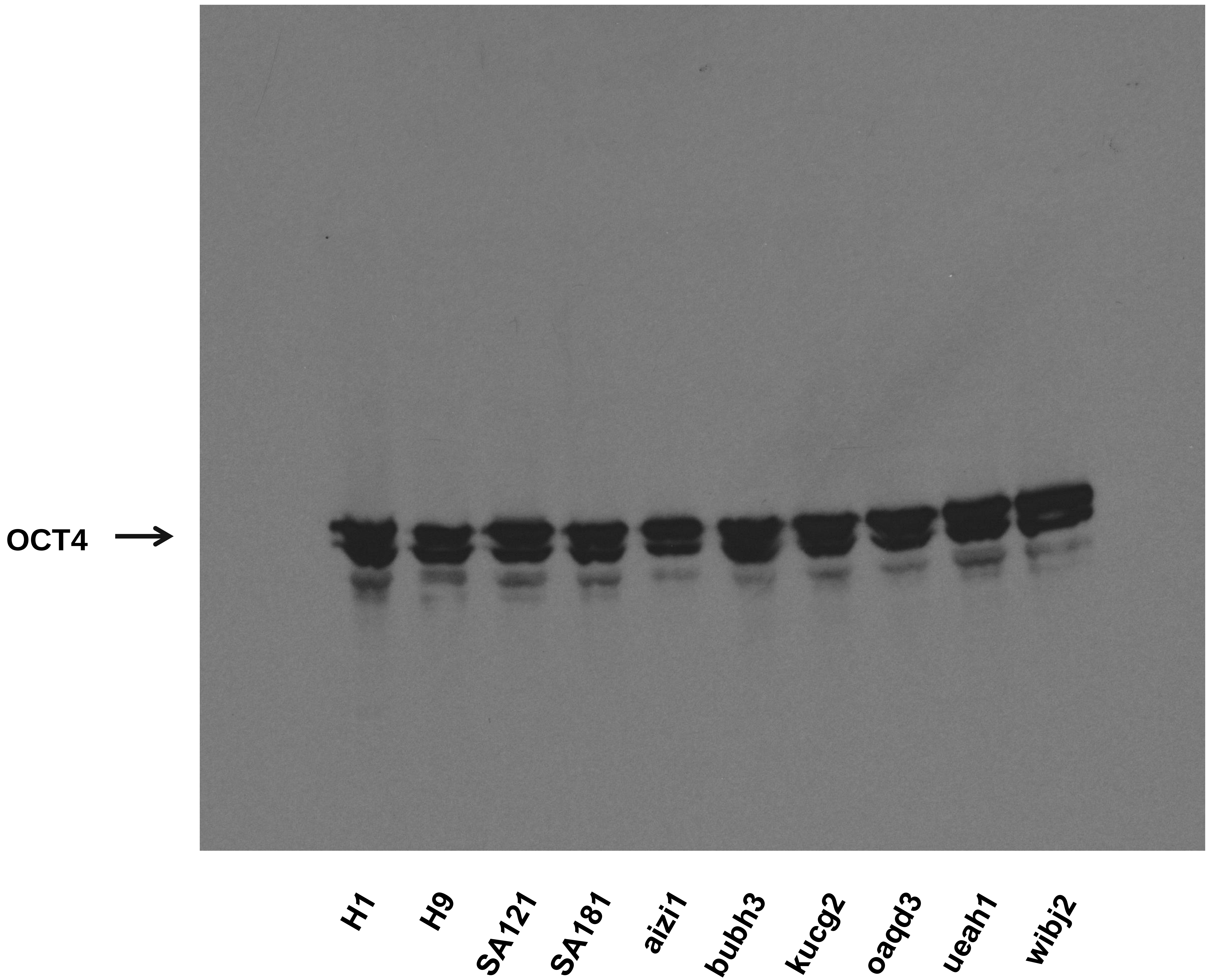
OCT4
H1
H9
SA121
SA181
aizi1
bubh3
kucg2
oaqd3
ueah1
wibj2

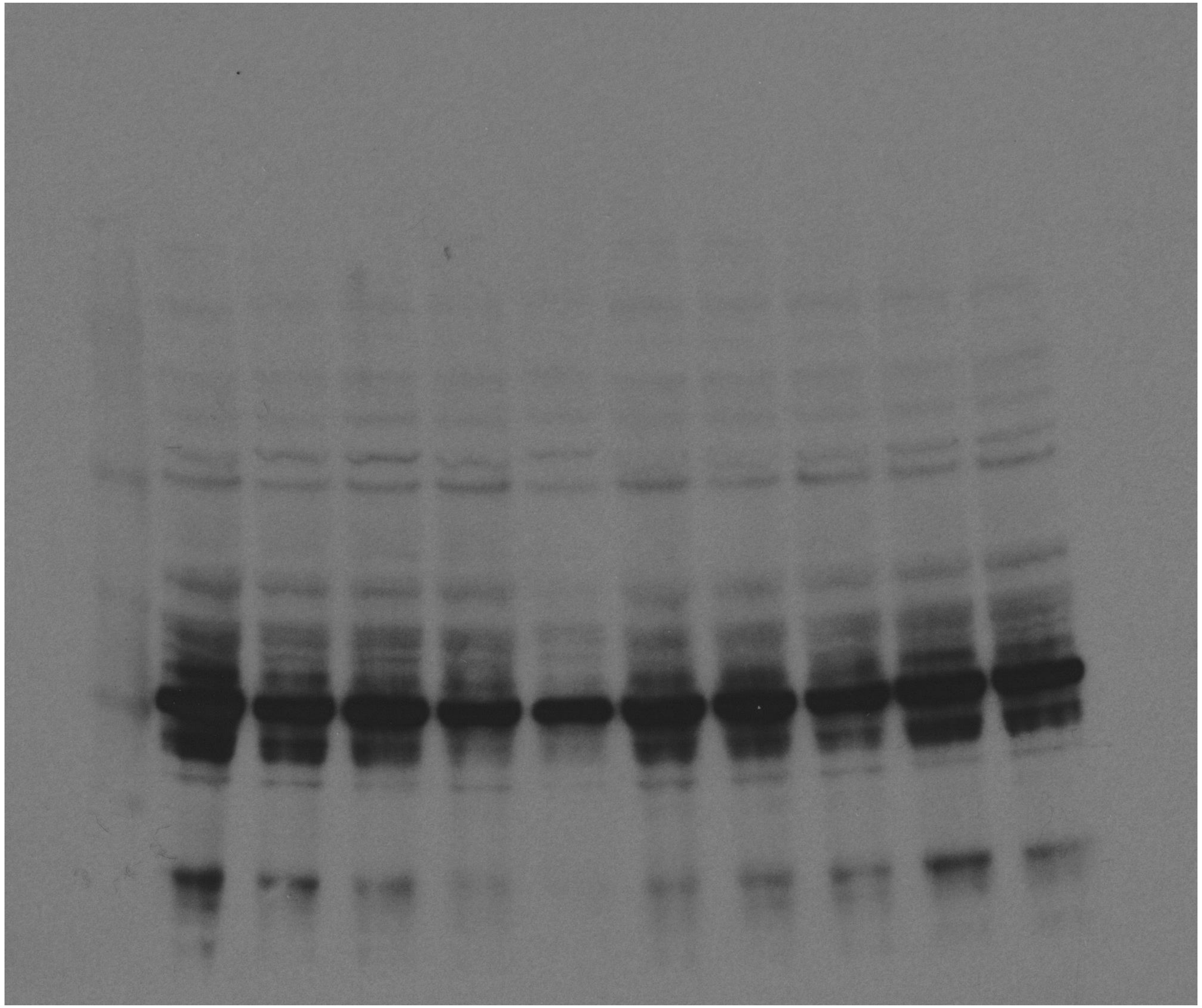

SOX2 →

H1 H9 SA121 SA181 aizi1 bubh3 kucg2 oaqd3 ueah1 wibj2

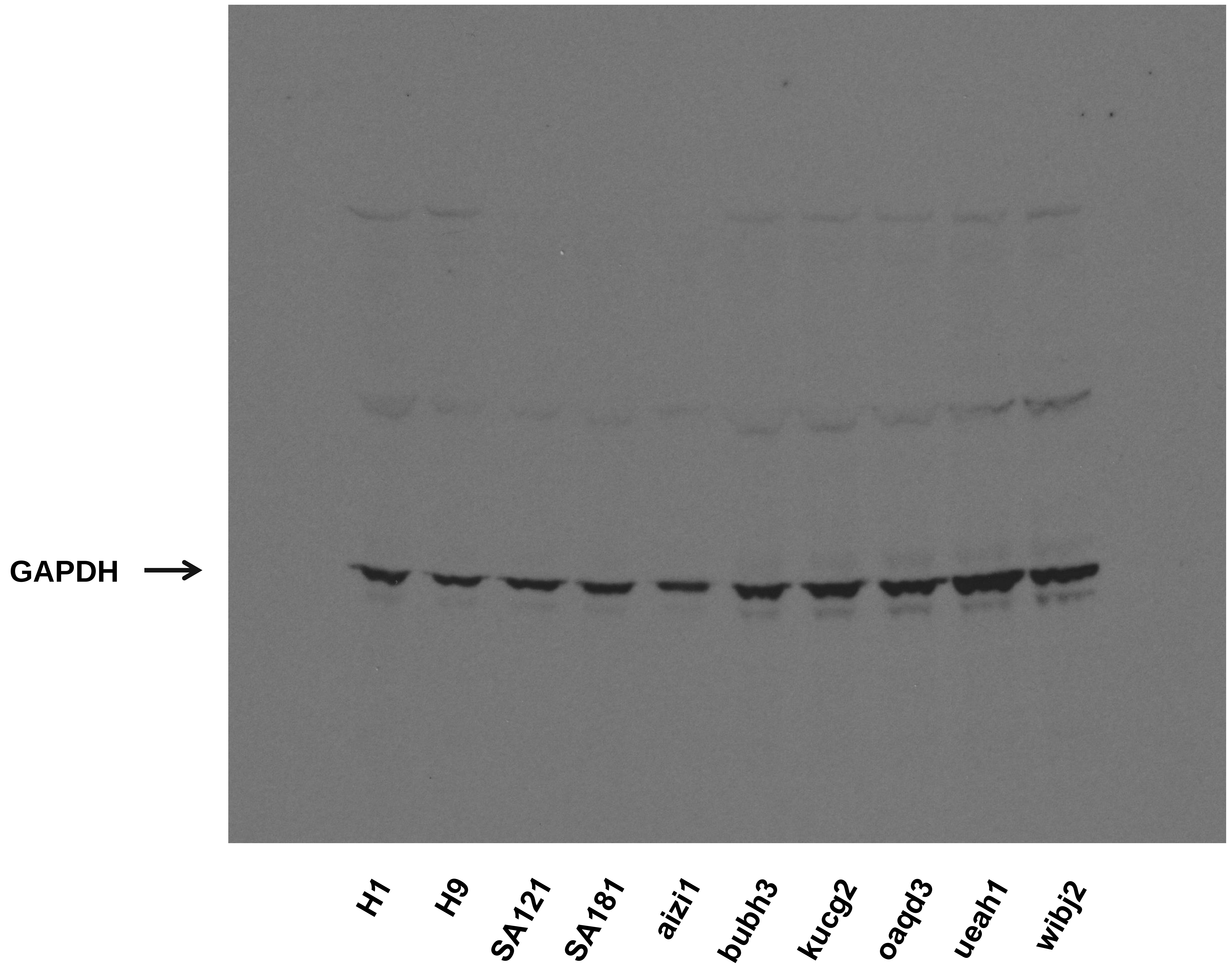

GAPDH
H1
H9
SA121
SA181
aizi1
bubh3
kucg2
oaqd3
ueah1
wibj2

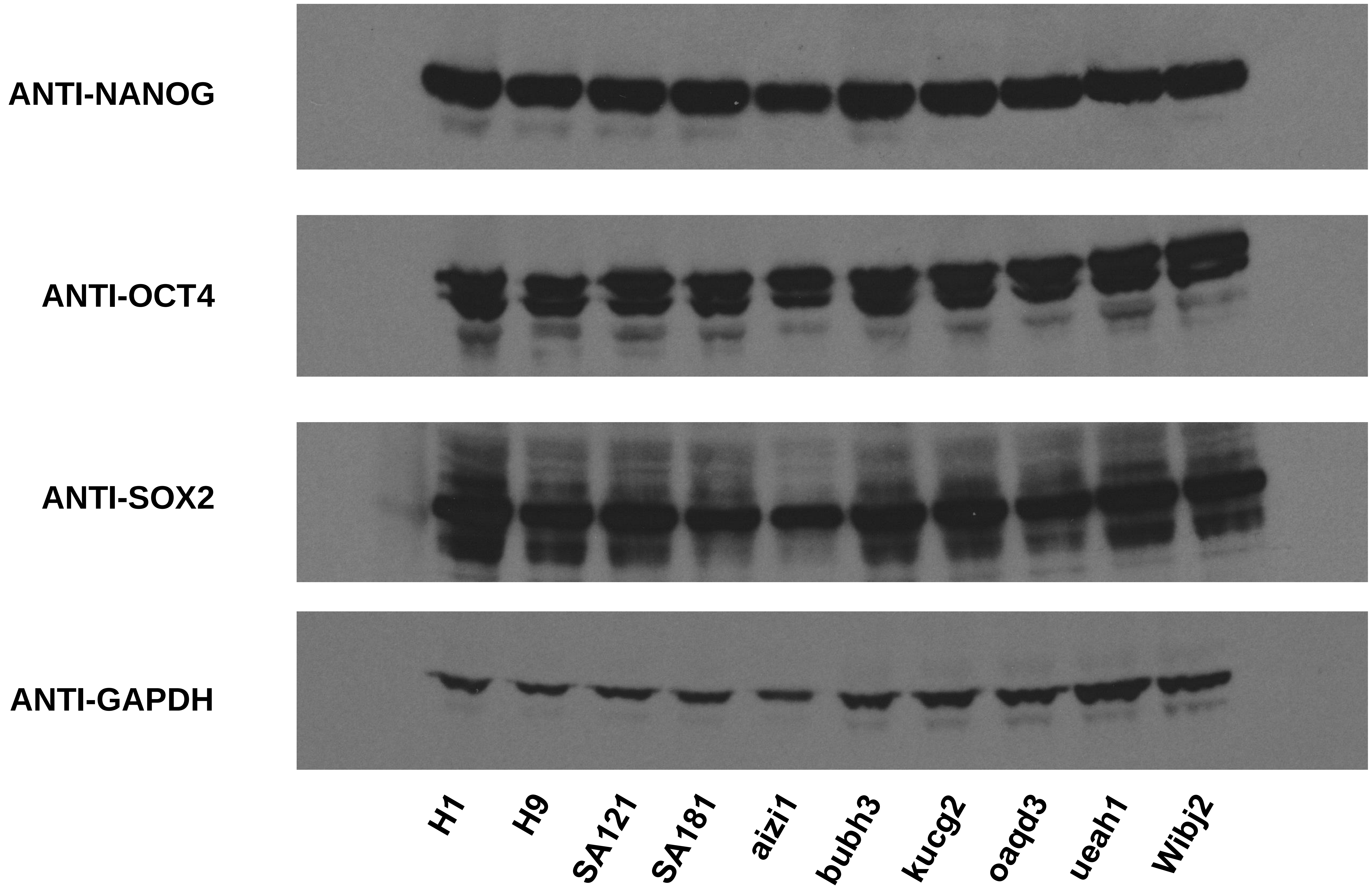
ANTI-NANOG
ANTI-OCT4
ANTI-SOX2
ANTI-GAPDH
H1
H9
SA121
SA181
aizi1
bubh3
kucg2
oaqd3
ueah1
Wibj2
